# Bladder Metastasis of Gastric Adenocarcinoma: Teaching point: Metastatic involvement has to be considered in the differential diagnosis for diffuse bladder wall thickening

**DOI:** 10.5334/jbsr.1765

**Published:** 2019-04-04

**Authors:** Ralph Khoury, Cristina Dragean, Laurence Annet

**Affiliations:** 1 UCL, Saint Luc Bruxelles, BE

**Keywords:** Bladder, Metastasis, gastric adenocarcinoma

## Case

A 75-year old man presented to his oncologist with a loss of appetite and a left lumbar pain. He was diagnosed two years earlier with focal adenocarcinoma of the pyloric region of the stomach and subsequently underwent partial gastrectomy.

Blood analysis showed renal insufficiency with glomerular filtration rate down to 36 ml/min/1.73 (compared to 85 ml/min/1.73, 6 months earlier).

**Figure 1:**
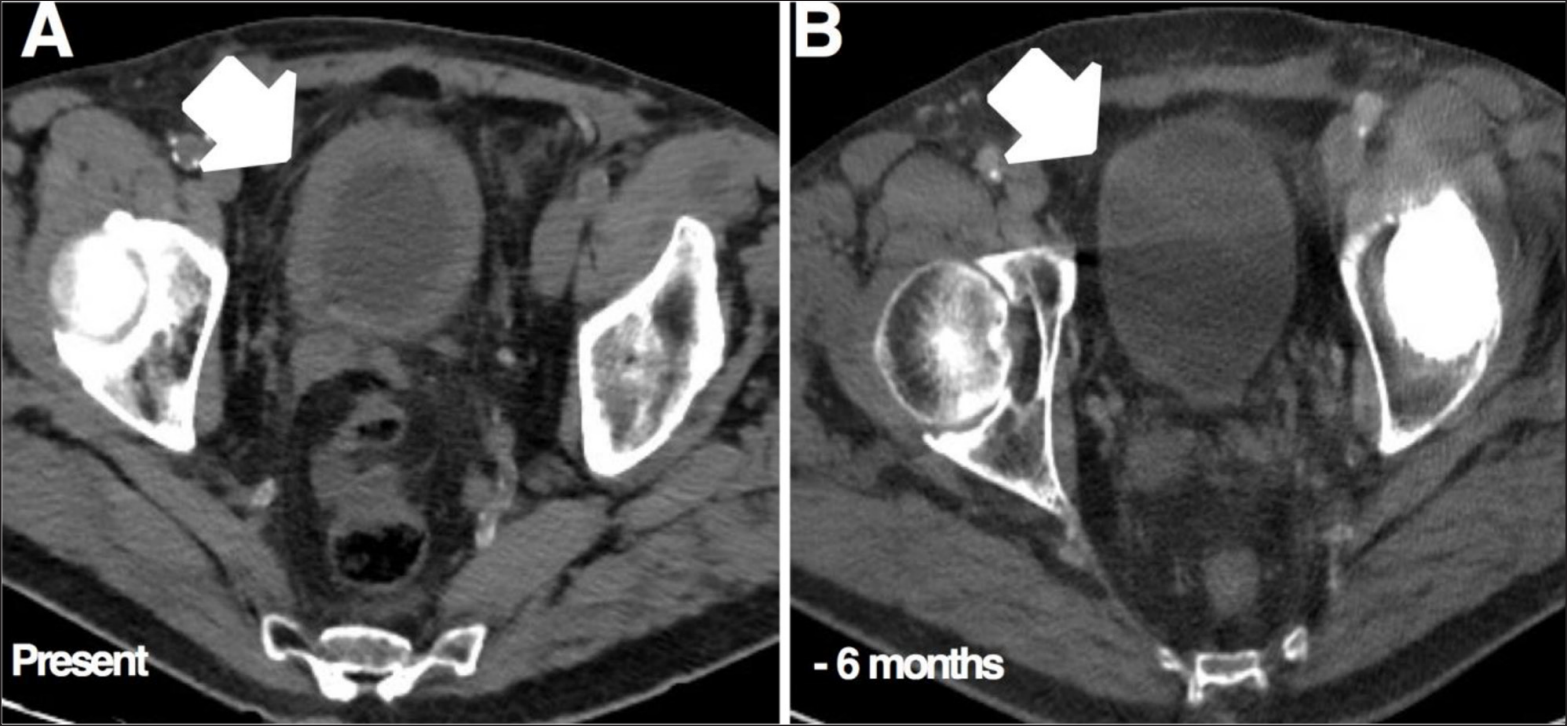


A non-contrast computed tomography (CT) was performed, showing bilateral hydronephrosis and a diffuse thickening of the bladder wall (Figure 1A) that was not present six months earlier (Figure 1B).

**Figure 2:**
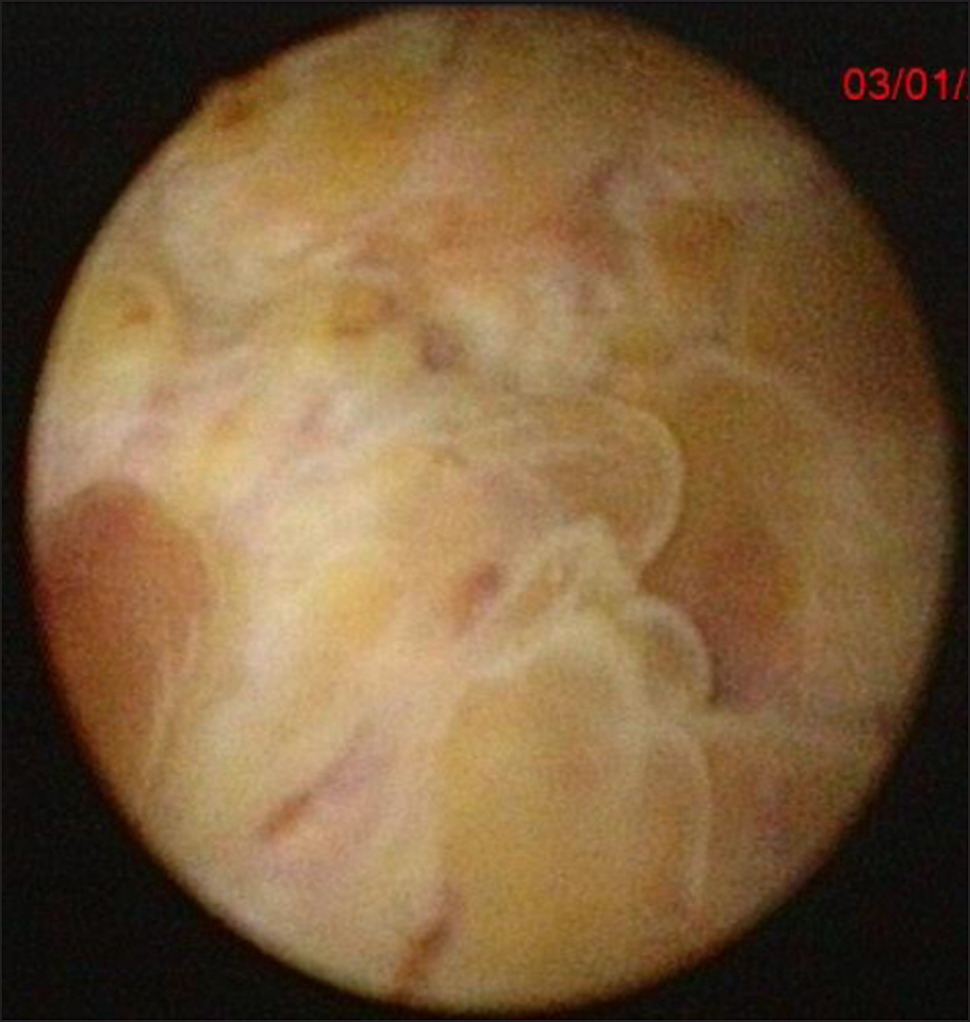


Subsequently, cystoscopy and biopsy were performed, showing an extensive vegetative lesion in the bladder (Figure 2).

**Figure 3:**
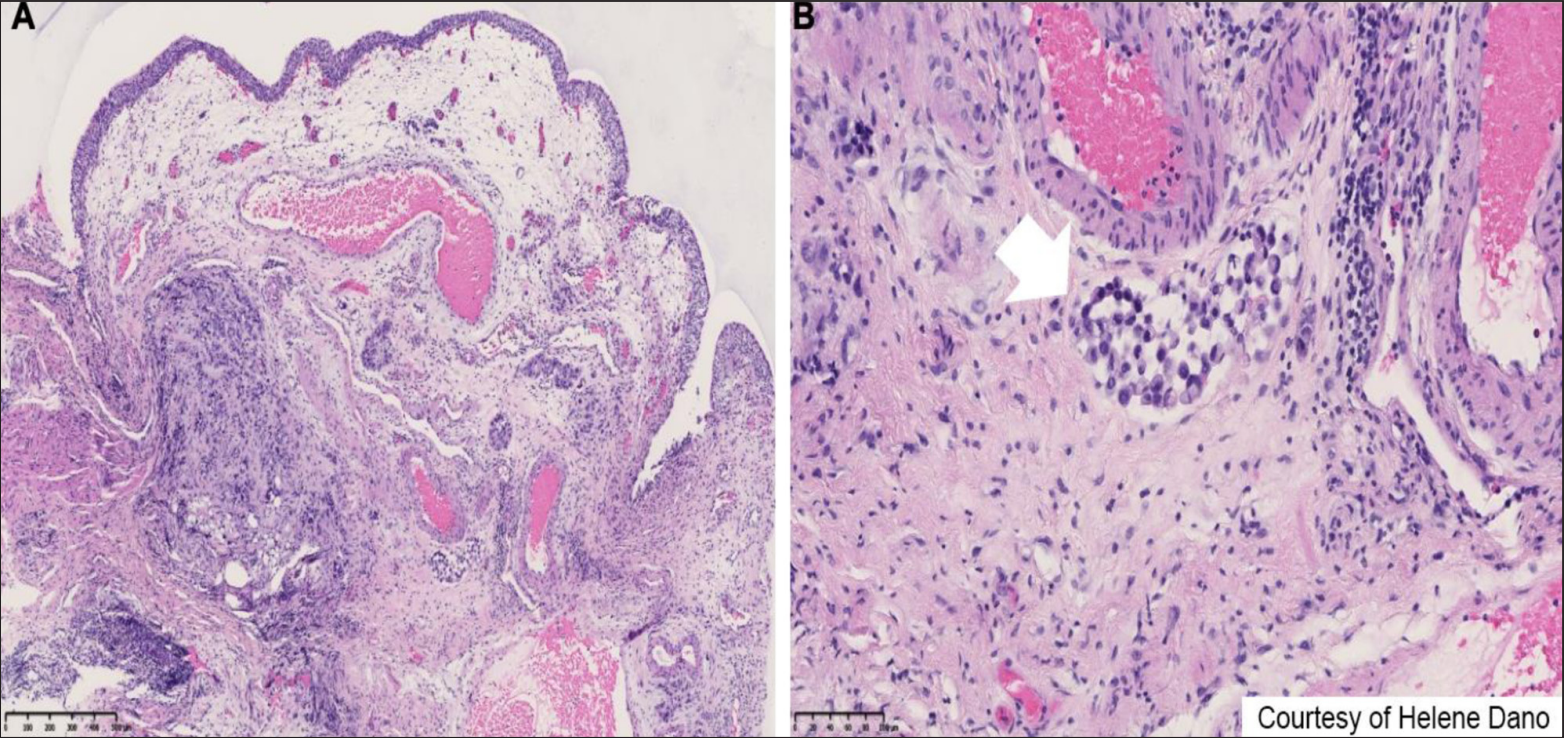


Histopathology showed a preserved epithelium with infiltration of the chorion, lymphatic permeation (Figure 3A), and signet ring cells (Figure 3B). While signet ring cells can be found in a number of tissues, they are most frequently associated with stomach cancer. In addition, immunostains of GATA3 and p40, which are sensitive markers for the differential diagnosis of bladder tumors, were negative, thus confirming an adenocarcinoma of gastric origin.

## Discussion

Bladder metastases are a rare entity, representing less than 2% of bladder tumors. Metastatic bladder tumors originate most commonly from melanoma, breast cancer, and gastric cancer. There are only few reported cases of bladder metastasis of gastric cancers in the literature (around 16) [1].

When we are confronted with bladder wall thickening, differential diagnosis may be developed based on whether it is focal or diffuse. Focal thickening suggests transitional cell carcinoma (90% of primary bladder tumors) and other bladder neoplasms (lymphoma, for instance). In our case, the thickening was diffuse; therefore we had to consider in the differential diagnosis the most frequent patterns: bladder outlet obstruction, neurogenic bladder, infectious cystitis, and cystitis from radiation or chemotherapy.

Our patient never had radiation, nor did he exhibit clinical or biological signs consistent with cystitis or bladder outlet obstruction (absence of benign prostatic hypertrophy). The patient had 5-fluorouracil/oxaliplatin (FOLFOX) chemotherapy in 2016; however, bladder toxicity is not associated with this therapy. Therefore, since the patient had none of these features, we suspected a bladder metastasis which was confirmed on histopathology.
